# Procedure for Femoral Intertrochanteric Fractures using the “Three‐Finger Method” Assisted by Proximal Femoral Nail Antirotation

**DOI:** 10.1111/os.12656

**Published:** 2020-04-28

**Authors:** Qian Cheng, MD, Li Lin, BM, Xiao‐dong Zhu, MD, Gui‐zhu Li, MD, Xiao‐ming Gao, BM, Yao Qian, BM, Guo‐yang Zhao, MD, Dong‐hua Di, MD

**Affiliations:** ^1^ Department of Orthopaedic, Tong Ren Hospital Shanghai Jiao Tong University School of Medicine Shanghai China; ^2^ Department of Orthopaedic, Affiliated Hospital of Jiangsu University Zhenjiang China; ^3^ Medical College, Jiangsu University Zhenjiang China

**Keywords:** Femoral intertrochanteric fracture, Long‐term efficacy, PFNA, Three‐finger method

## Abstract

**Objective:**

To assess long‐term follow‐up evaluations for the treatment of femoral intertrochanteric fractures with the “three‐finger method” assisted by proximal femoral nail antirotation (PFNA).

**Methods:**

From January 2010 to January 2017, 123 patients were selected and followed for the treatment of femoral intertrochanteric fractures with PFNA assisted by the “three‐finger method” (application of the index finger, middle finger, and ring finger in the process of surgery to assist PFNA). There were 56 male patients and 67 female patients aged 52–93 years with an average age of 75.6 years, and 88 cases were due to a fall and 35 due to a traffic accident injury. The femoral necks were fixed with PFNA assisted by the “three‐finger method” applying the following procedure: traction reduction, determining the incision, inserting the needle, and placing screw. The Harris hip score, postoperative complications, hip pain and function status were statistically analyzed to evaluate the surgical efficacy and to discuss the surgical technique of the “three‐finger method” assisted by PFNA.

**Results:**

According to the Harris scoring criteria, patients were followed for 1, 2, 4, 6, and 8 years, and the good outcomes of patients were recorded. The excellent and good rate of 87% was the highest in the second year of follow‐up. Then, the rate decreased following the eighth year of follow‐up. The excellent and good rate of 82.7% was the lowest. The patients with incisions healed well, there were no instances of fat liquefaction or infection. There were three cases of effusion, the rate was 2.4%. The secretions were cultured, and no bacterial growth was found. After treatment of the wound, it healed, and the spiral blade used for the femoral head did not wear out. There was one case of femoral head necrosis. There was no significant correlation between hip pain and sex and age (*P* > 0.05), and the function of the hip joint was significantly correlated with the age of the patients (*P* < 0.05).

**Conclusion:**

The “three‐finger method” in the process of surgery to assist PFNA for the treatment of patients with intertrochanteric fractures of the femur simplified the operation steps, reduced the operation difficulty, shortened the operation time, improved the operation efficiency, and reduced the incidence of postoperative complications.

## Introduction

Hip fractures represent a major public health problem for older adults compounded by the problems of aging^1^. The annual incidence of hip fractures is increasing rapidly and is projected to surpass 6.3 mn by 2050^2^. The situation is even worse in China. There are approximately 230,000 new hip fractures in China each year. It is expected that 50% of the world's new hip fractures will occur in China by 2050^3^. Regarding the elderly population, hip fractures can be fatal, with 1‐year mortality rates ranging from 14% to 36%. Approximately 50% of survivors are disabled and unable to take care of themselves, and their quality of life is significantly reduced^4^, ^5^. Their treatment consumes a growing percentage of healthcare expenditures, and hip fractures were ranked the 13th most expensive diagnoses by Medicare in 2011, with an estimated cost of up to $15 bn annually. Moreover, a proportion of patients with hip fractures may require placement in long‐term care facilities, and the need for this additional care and supervision following surgical treatment confers significant societal and personal economic burden^6^.

Hip fractures are anatomically classified in relation to the hip capsule as intracapsular fractures (i.e., at the femoral neck) or extracapsular fractures (i.e., intertrochanteric or subtrochanteric fractures)^7^. Intertrochanteric fractures (ITFs) are one of the most common types of hip fractures, accounting for 45%–50% of total hip fractures, of which 35%–60% are unstable fractures^8^. Intertrochanteric fractures in the elderly population are mostly osteoporotic fractures in the form of a comminuted fracture in which the bone has broken into 3–4 fragments in 30%–40% of cases^8^. The treatment for intertrochanteric fractures of the femur can be divided into conservative treatment and surgical treatment^9^. Most doctors agree that conservative treatment of ITFs results in serious complications and sequelae, such as the need for bed rest and limb traction, and a mortality rate within 1 year after injury is relatively high^10^. Surgical treatment achieves firm and stable internal fixation of the fracture and early functional training, and reduces complications due to long‐term bed rest^11^, ^12^. However, fixation failure or nonunion occasionally occurs, commonly in patients with unfavorable fracture patterns, unsatisfactory reduction quality, poor bone quality, inappropriate choice of internal fixation device, or improper implant position^13^, and surgical intervention is recommended in patients with these types of fractures with the use of various implants. The fixation options for intertrochanteric fractures include intramedullary or extramedullary fixation^14^. For stable intertrochanteric fractures, there is currently little evidence in terms of the superiority of one device over another for the management of these fractures, and the quality of reduction remains paramount. For unstable intertrochanteric fractures, an intramedullary device is recommended according to current guidelines^15^. The success of internal fixation for intertrochanteric fractures in elderly patients mainly depends on the severity of the osteoporosis, fracture type, fixator position, and patient compliance^16^. Intramedullary nails with cephalomedullary screws or a sliding hip screw‐plate construct are the standard surgical treatment options chosen by most surgeons^17^, ^18^, ^19^. Over the past decades, the dynamic hip screw (DHS) as an extramedullary stabilization device has been the most widely used implant; this type of screw has the advantages of being easy to use and having a low cost, a low amount of blood loss, a lower reoperation rate, and good functional outcomes^16^, ^20^. Clinical studies show that the DHS is associated with a lower incidence of femoral fracture and a lower reoperation rate than the intramedullary nail, especially for stable intertrochanteric fractures^16^. However, with unstable fractures, complications such as hip varus deformities and delayed union often occur with DHS fixation^21^. Therefore, some studies have suggested that intramedullary devices may be highly effective for the internal fixation of unstable intertrochanteric fractures and that the DHS should be implemented with caution due to its associated high complication rates and poor functional outcomes^5^, ^22^.

Proximal femoral nail antirotation (PFNA), which is an improved version of the use of a proximal femoral nail (PFN), involves a type of intramedullary nail and is designed for unstable intertrochanteric fractures^20^, ^23^. According to Sharma and Mahajan, PFNA has a superior performance over PFN in the setting of osteoporosis, which is attributed to compaction of cancellous bone by the helical blade^24^. The main features of PFNA are the introduction of a 6.5 mm antirotation neck screw, the fluting of the nail tip to decrease stress, and the more proximal positioning of the distal locking screws to avoid abrupt changes in the stiffness of the construct^25^. The helical blade is said to increase the bone‐implant interface and result in the compaction of cancellous bone, thereby providing excellent stability during fixation. Compared with the DHS, PFNA is characterized by minimal invasiveness, decreased operation durations, and accelerated postoperative recovery in the treatment of osteoporotic intertrochanteric fractures^26^.

Despite the fact that complications, including anterior thigh pain and implant failure/secondary fracture, with a reported incidence rate of approximately 2.0%–3.5%, seriously affect patients' quality of life^27^, the treatment of femoral intertrochanter fractures with PFNA has been approved by domestic and international doctors. However, long‐term follow‐up studies on the efficacy of PFNA have not been reported. In this study, 123 patients with femoral intertrochanteric fractures who were treated with PFNA assisted by the “three‐finger method” in our hospital were followed for a long period of time. The purpose of this investigation was as follows: (i) to consider a new surgical method of proximal femoral nail antirotation for intertrochanteric fractures assisted by the “three‐finger method” as evaluated by the Harris hip score, the incidence of postoperative complications, the incidence of postoperative hip pain, and the function of the hip joint; (ii) to explore whether there is a relationship between some postoperative complications and age; and (iii) to discuss the superiority of this surgical method.

## Materials and Methods

### *Inclusion and Exclusion Criteria*

#### *Inclusion Criteria*

The inclusion criteria following PICOS principle were as follows: (i) patients who were at least 50 years old with intertrochanteric fractures; (ii) patients who had undergone proximal femoral nail antirotation assisted by the “three‐finger method”; (iii) comparison was performed between different ages and genders; (iv) the Harris hip score, the incidence of postoperative complications, the incidence of postoperative hip pain, and the function of the hip joint were evaluated; and (v) patients were followed‐up for at least 1 year.

#### *Exclusion Criteria*

The exclusion criteria were as follows: (i) pathological fractures (such as bone metastasis, primary bone tumor, and metabolic bone disease); (ii) history of fractures in the affected hip; (iii) other types of hip fractures; (iv) arthritis or femoral head necrosis in the affected side of the hip; and (v) the postoperative follow‐up time was less than 1 year.

### *General Information of the Participants*

A total of 123 patients admitted to our hospital from January 2010 to January 2017 were selected and followed for the treatment of femoral intertrochanteric fractures with PFNA assisted by the “three‐finger method”; there were 56 male patients and 67 female patients aged 52–93 years old with an average age of 72.7 ± 12.0 years; 88 cases were due to a fall and 35 due to a traffic accident injury. According to Evans, 18 patients had type II fractures, 46 patients had type III fractures, and 59 patients had type IV fractures. Before the operation, 59 patients had hypertension, 25 patients had diabetes, and 20 patients had the sequela of cerebral infarction (with normal limb muscle strength); there were also nine patients with cardiac dysfunction and arrhythmia.

### *Surgical Strategy and Procedures*

The “three‐finger method” refers to the application of the index finger, middle finger, and ring finger during the surgical process for assisting PFNA in the treatment of patients with intertrochanteric fractures of the femur; this method simplifies the operation steps and makes the operation convenient and fast.

#### *Anesthesia and Position*

**Figure 1:**
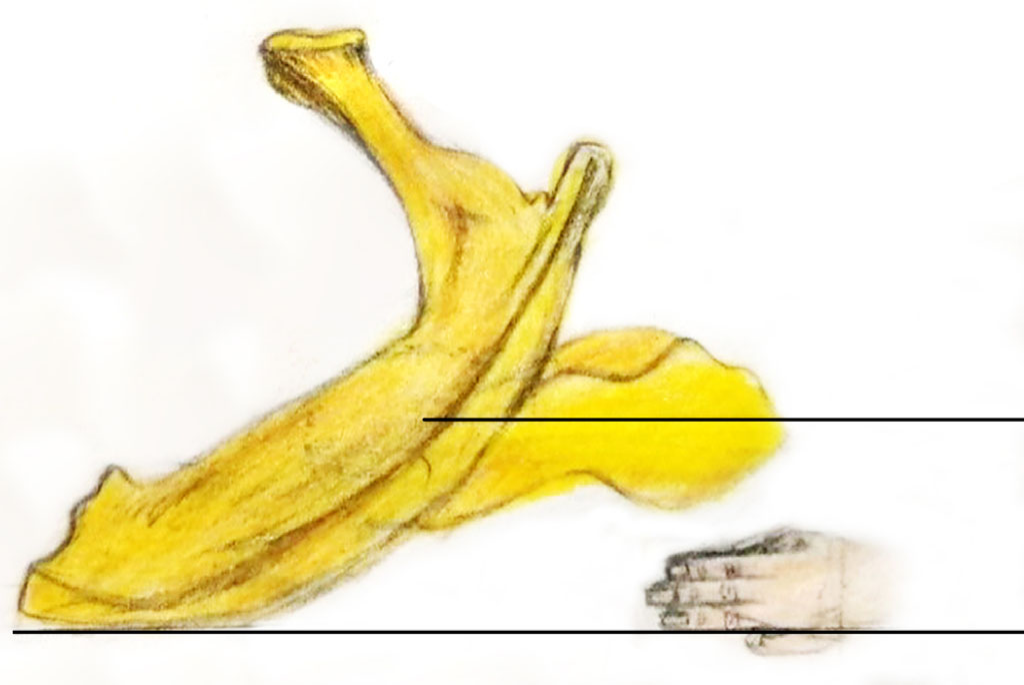
The injured hip and the three fingers used; the height of the three fingers was approximately 4–5 cm.

During the surgical procedures, we used epidural, lumbar, or general anesthesia. After successful anesthesia, the patient was placed in a traction bed with the pelvis in a horizontal position. Figure 1 shows the injured hip and the three fingers used, and the height of the three fingers was approximately 4–5 cm.

#### *Fracture Reduction and Traction*

We conducted routine disinfection of and placed sterile surgical towels in the operation area. With the use of the C‐arm of the X‐ray machine, traction reduction of the fracture was performed, and the anterior and posterior positions and lateral positions of the fracture were examined.

#### *Approach and Exposure*

**Figure 2:**
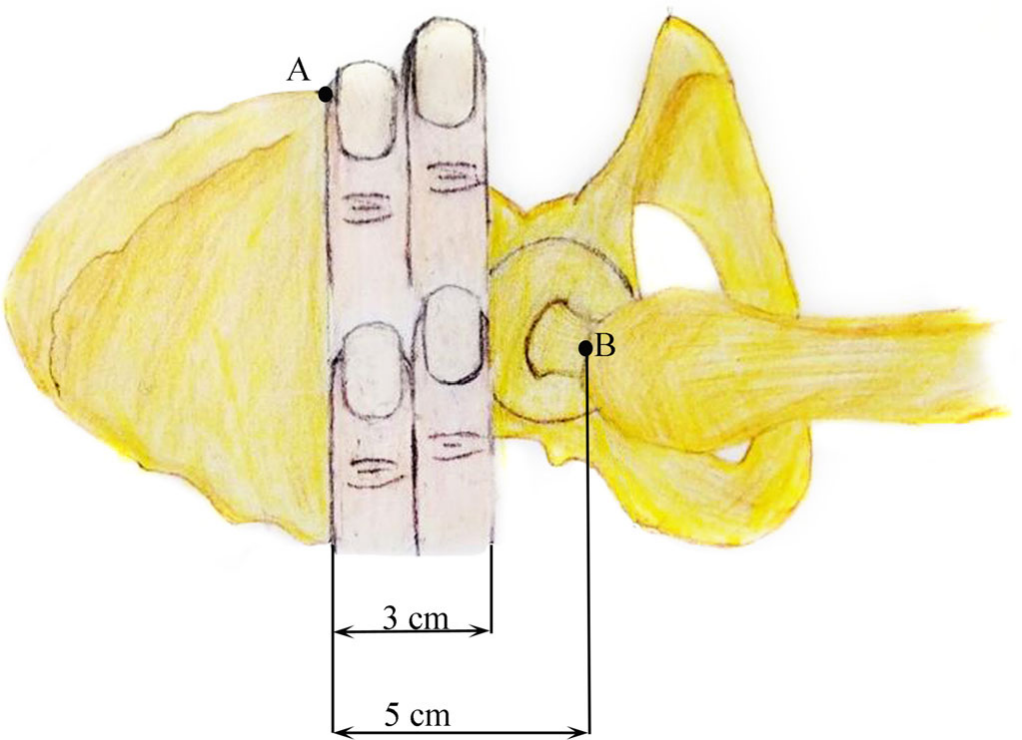
We used two fingers to determine the incision position; the index finger indicates the anterior superior iliac spine and runs backward vertically along the longitudinal axis of the femoral shaft. A Longitudinal incision, approximately 3 cm in length, was made at the width of the two fingers. (A) Anterior superior iliac spine, (B) top of the greater trochanter.

After satisfactory reduction, we used two fingers to determine the incision position (Fig. 2). With the two fingers placed side‐by‐side, the index finger touched the anterior superior iliac spine and moved backward vertically along the longitudinal axis of the femoral shaft. A longitudinal incision, approximately 3 cm long, was made at the width of the two fingers, and the skin, subcutaneous tissue, and tensor fasciae latae were cut in succession, followed by blunt separation of the muscle tissue.

#### *Insertion and Fixation*

**Figure 3:**
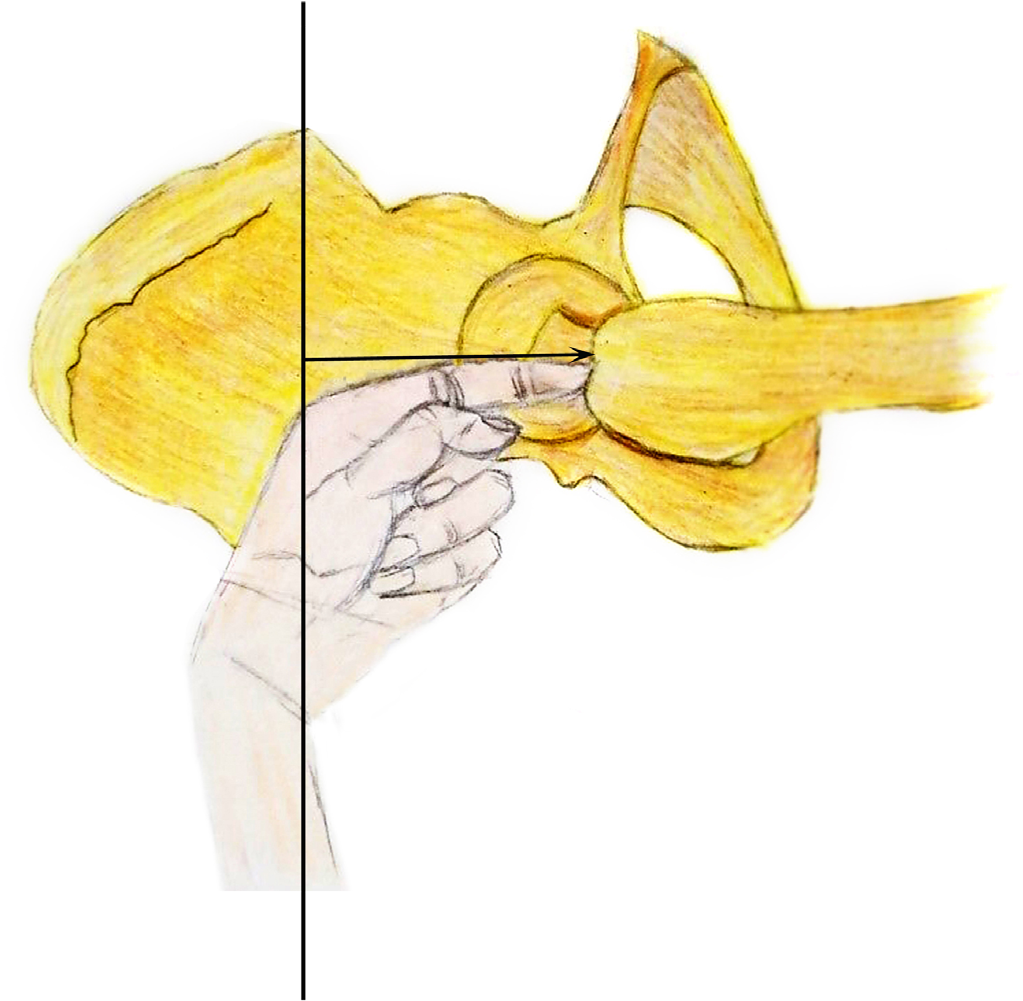
We used one finger to determine the position of the insertion point. The index finger was extended from the incision and placed on the inside of the highest point of the greater trochanter. The insertion point was close to the front of the index finger, indicating the medial one‐third of the anterior peak of the greater trochanter; we inserted the needle here.

We then used one finger to determine the position of the insertion point (Fig. 3). The index finger was extended from the incision and placed on the inside of the highest point of the greater trochanter. The insertion point was close to the front of the index finger and represented the medial one‐third of the anterior peak of the greater trochanter. We inserted the needle and opened the cavity with a medullary drill; according to the width of the medullary cavity, we chose appropriate PFNA, inserted the guide needle, and viewed the anterior, posterior, and lateral positions.

#### *Reconstruction*

According to the depth of the guide needle, an antirotation blade of corresponding length was inserted into the neck of the femur. The tip of the blade was located 0.5–1 cm below the femoral head. Finally, we screwed in a distal locking pin. The wound was subsequently rinsed, the subcutaneous layer and skin were sutured, and the surgery was completed.

### *Postoperative Treatment*

Postoperative antibiotics and low‐molecular‐weight heparin anticoagulant therapy were used to prevent infection. Limb muscle massages were administered postoperatively; at the same time, patients were encouraged to perform activities and exercises of ankle plantar flexion, active back stretching, and isotonic quadriceps contraction. These activities can help eliminate swelling, promote venous reflux, and prevent deep venous thrombosis. X‐rays were reexamined 3 days after surgery. After 3 to 4 weeks, the patients could use both crutches to stand without weight‐bearing of the affected limb. After 6 to 8 weeks, partial weight‐bearing activity was initiated for the affected limb, and after 12 weeks, the patients could walk fully on the affected limb with weight‐bearing.

### *Postoperative Follow‐up and Statistical Treatment*

Within half a year after the surgery, we performed a regular outpatient review of each patient, and we reviewed the healing of the fracture and checked the recovery of hip joint function. After six months, telephone follow‐up was conducted to help guide the rehabilitation exercises. According to the Harris scoring standard of the hip joint, the relevant data were recorded according to the follow‐up times of 1, 2, 4, 6, and 8 years.

### *Observation Indicators*

#### *Harris Hip Score (HHS)*

The HHS was used to evaluate the postoperative recovery of hip function. The HHS system mainly includes four aspects: pain, function, absence of deformity, and range of motion^28^.

We calculated the score 1, 2, 4, 6, and 8 years after the operation to obtain a rough measurement of hip function. The score standard has a maximum of 100 points (best possible outcome). A total score <70 is considered poor, 70–79 is considered general, 80–89 is considered good, and 90–100 is considered excellent.

#### *Incidence of Postoperative Complications*

Postoperative complications include infection, varus malunion, internal fixation fracture or loose and secondary fractures. All the patients were followed to observe whether postoperative complications occurred. We then calculated the incidence of postoperative complications.

#### *Incidence of Postoperative Hip Pain*

Hip pain is a sign of hip function and can directly affect patients' quality of life. All the patients were followed to determine whether hip pain existed, and we calculated the rate. Furthermore, we grouped all the patients based on age and sex to observe whether there was any relationship between hip pain and age or sex.

#### *Function of the Hip Joint*

According to the HHS, we chose the three activities of climbing stairs, walking, and putting on socks, as these are the most basic and common activities in the elderly population, to evaluate the function of the hip joint. We calculated the difficulty rate of performing these three activities and compared them by age.

### *Statistical Analysis*

The obtained data were analyzed with SPSS 18.0 statistical software (International Business Machines Corporation, Armonk, New York, USA), and the *t*‐test was used for the comparison of HHS between the groups. The count data, including rate of postoperative complications and hip pain and function of the hip joint, are represented by the rate of the test, which was measured by the χ^2^ test, with a significant difference of *P* < 0.05.

## Results

### *General Results*

The longest hospitalization length was 30 days, the shortest length was 5 days, and the average length of hospitalization was 15.2 ± 8.3 days. Surgical treatment was performed within 2 weeks after admission, with an average of 6.6 ± 3.0 days of hospitalization before surgery. The longest follow‐up time was 8 years, and the shortest follow‐up time was 1 year, with an average of 4.2 ± 1.8 years. The average operation time was 89.2 ± 25.3 min (range, 45–180 min). The average blood loss during the operation was 312.7 ± 145.3 mL (range, 150–700 mL). In the progress of inserting the needle with the help of index finger, attention must be paid to avoid piercing rubber gloves, as hands with the rubber gloves are not very sensitive to touch.

### *HHS*

Among the 123 patients, there were 25 deaths, the mortality rate was 20.3%, and the cause of death was due to internal medicine diseases. In 10 patients, the fractures healed after surgery, PFNA was removed; the average removal time was 18 months after surgery.

According to the Harris scoring criteria of the hip joint, patients were followed for 1, 2, 4, 6, and 8 years, and their conditions were recorded (Table 1). In the second year of follow‐up, the excellent and good rate was the highest (88.7%); then, it decreased following the eighth year of follow‐up. The lowest best and good rate was 82.7%.

**Table 1:** Harris hip score

Follow‐up time (years)	Excellent (cases)	Good (cases)	General (cases)	Bad (cases)	Death (cases)	Total (cases)	Excellent‐good‐rate (%)
1	57	43	19	3	1	123	82
2	71	31	11	2	8	123	88.7
4	67	25	14	3	14	123	84.4
6	61	28	12	5	17	123	83.4
8	56	25	11	6	25	123	82.7

### *Incidence of Postoperative Hip Pain*

**Figure 4:**
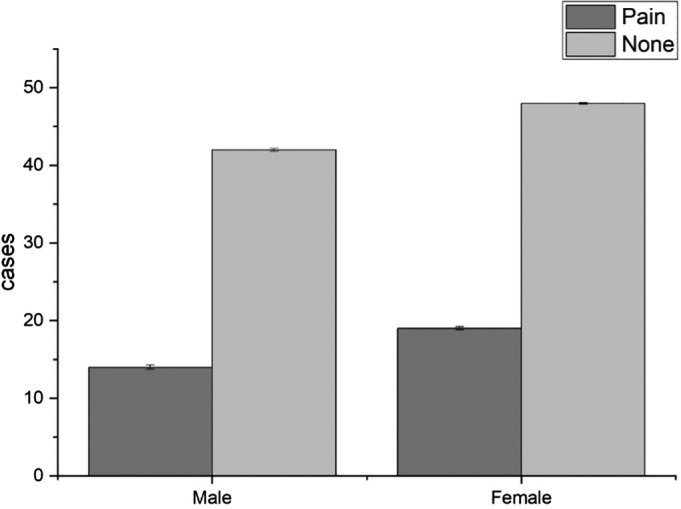
Relationship between pain and sex. From the follow‐up results of 123 patients, we found that the incidence of hip joint pain was 26.8%: 25.0% were males and 28.4% were females; there was no significant relationship between the incidence of hip pain and sex (*P* > 0.05).

**Figure 5:**
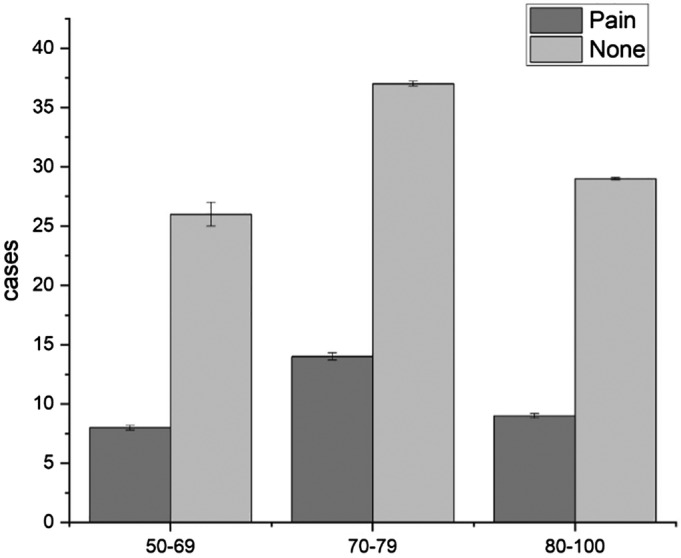
Relationship between pain and age. From the follow‐up of 123 patients, there were 34 patients between 50 and 69 years old and 8 patients with pain, and the incidence of pain was 23.5%. There were 51 patients between 70 and 79 years old and 14 patients with pain, and the incidence of pain was 27.5%. There were 38 patients between 80 and 100 years old and nine patients with pain, and the incidence of pain was 23.7%. The incidence of hip pain was not significantly associated with age (*P* > 0.05).

During the follow‐up of 123 patients, we found that the incidence of hip joint pain was 26.8%: of these, 25.0% were males and 28.4% were females (Fig. 4). The patients were divided into three groups according to age. Among them, there were 34 patients between 50 and 69 years, eight of which had pain, and the incidence of pain was 23.5%. There were 51 patients between 70 and 79 years old, 14 of which has pain, and the incidence of pain was 27.5%. There were 38 patients between 80 and 100 years old, nine of which had pain, and the incidence of pain was 23.7% (Fig. 5). The incidence of hip pain was not statistically significant, and the pain in the hip joint was not correlated with sex or age (*P* > 0.05).

### *Function of the Hip Joint*

**Figure 6:**
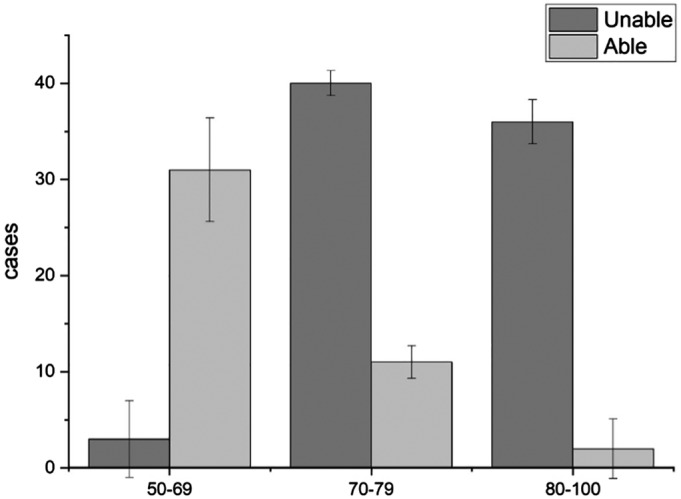
The relationship between the climbing stairs, walking and age. There were three patients in the 50–69‐year age group with difficulty walking and going up and down stairs, with an incidence of 8.6%. There were 40 patients in the 70–79‐year age group, with an incidence of 78.4%. There were 36 patients in the 80–100‐year age group, with an incidence of 94.7%. The function of the hip joint was significantly correlated with the age of the patient (*P* < 0.05).

**Figure 7:**
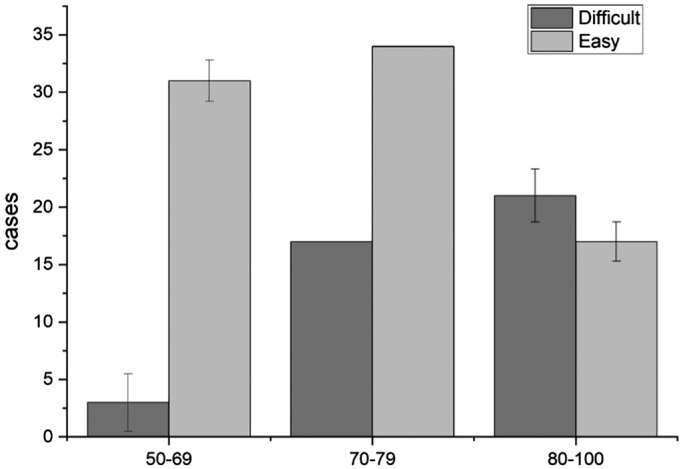
The relationship between wearing socks and age. There were three patients in the 50–69‐year age group, with an incidence of 8.6%. There were 17 patients in the 70–79‐year age group, with an incidence of 33.3%. There were 21 patients in the 80–100‐year age group, with an incidence of 55.3%. The function of the hip joint was significantly correlated with the age of the patient (*P* < 0.05).

A total of 123 patients were followed, and there were three patients in the 50–69‐year age group with difficulty walking and going up and down stairs, with an incidence of 8.6%. There were 40 patients in the 70–79‐year age group, with an incidence of 78.4%. There were 36 patients in the 80–100‐year age group, with an incidence of 94.7% (Fig. 6). There were three patients in the 50–69‐year age group, with an incidence of 8.6%. There were 17 patients in the 70–79‐year age group, with an incidence of 33.3%. There were 21 patients in the 80–100‐year age group, with an incidence of 55.3% (Fig. 7).

The function of the hip joint was significantly correlated with the age of the patient (*P* < 0.05). As the age of the patients increased, the capacity for action became poor and the walking distance became shorter; thus, the function of the hip joint worsened.

### *Postoperative Complications*

Of the 123 patients, there were no instances of fat liquefaction or infection, and three patients had effusion; the rate was 2.4%. The secretions were cultured, and no bacterial growth was found. The wound healed after treatment. In all patients with long‐term follow‐up, all fractures healed; there were no hip introversive deformities, cases of internal fixation without fracture or loose fixation, or secondary femoral fractures. One of the patients had femoral head necrosis 1 year after surgery, and the rate was 0.8%. After artificial femoral head replacement, the patient recovered well.

**Figure 8:**
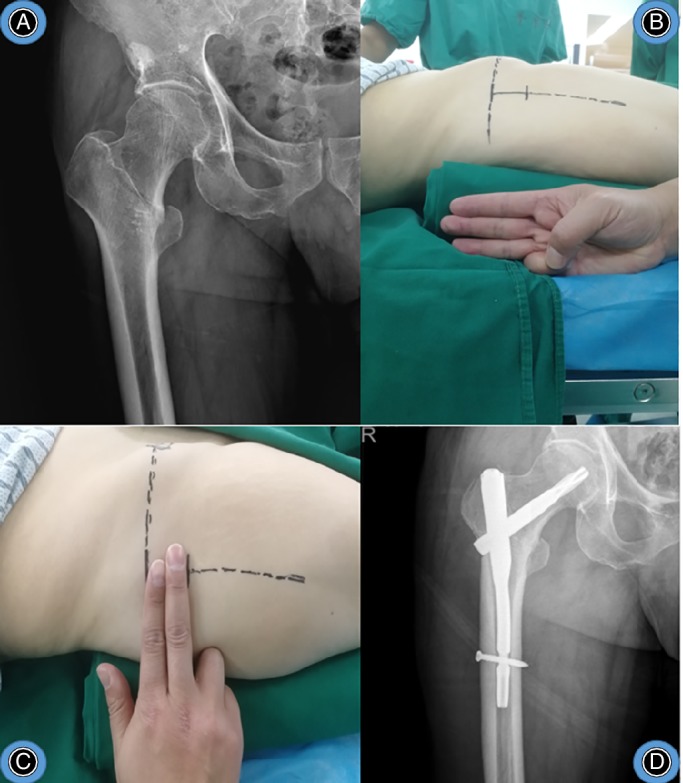
Female, 75 years old, crash injury, right intertrochanteric fracture, treated with “three‐finger method” assisted by PFNA. (A) preoperative X‐ray of right hip. (B) patient position of three fingers high. (C) incision position. (D) postoperative X‐ray of right hip.

**Figure 9:**
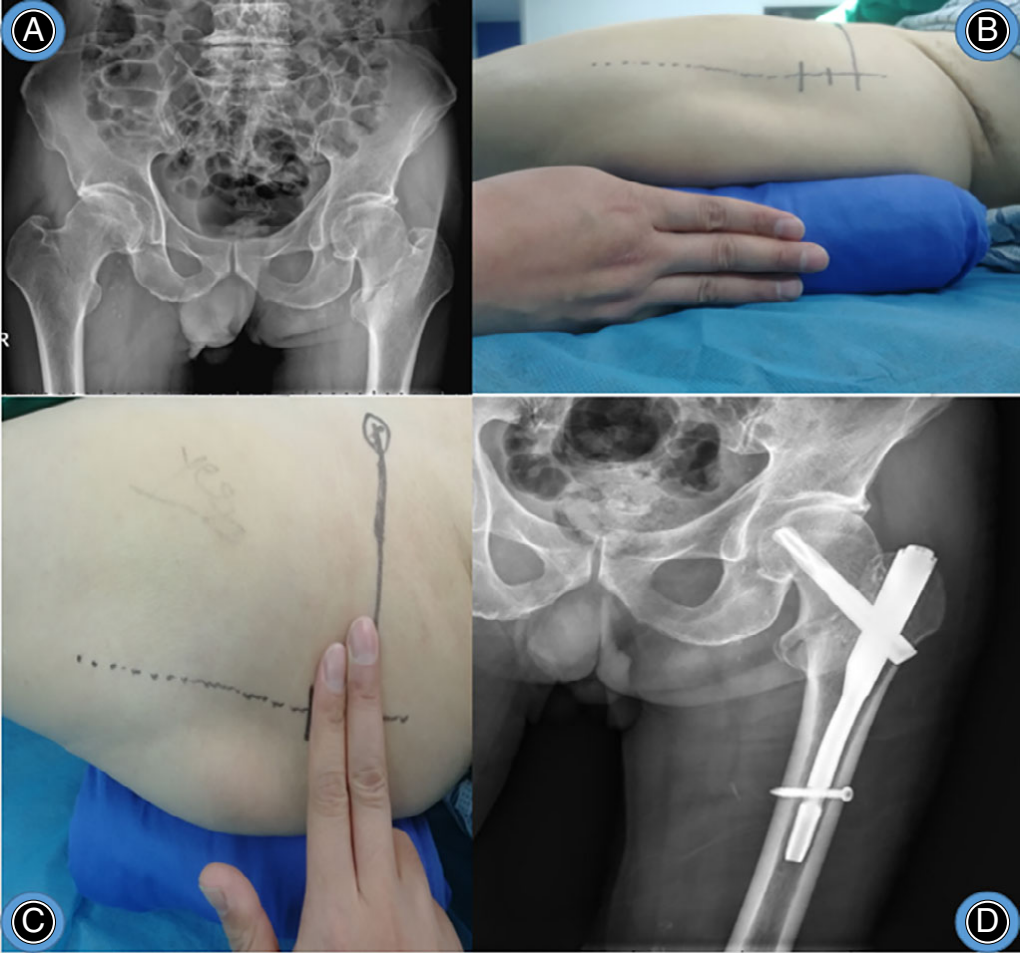
Male, 77 years old, fall injury, left intertrochanteric fracture, treated with “three‐finger method” assisted by PFNA. (A) preoperative X‐ray of pelvis. (B) patient position of 3 fingers high. (C) incision position. (D) postoperative X‐ray of left hip.

**Figure 10:**
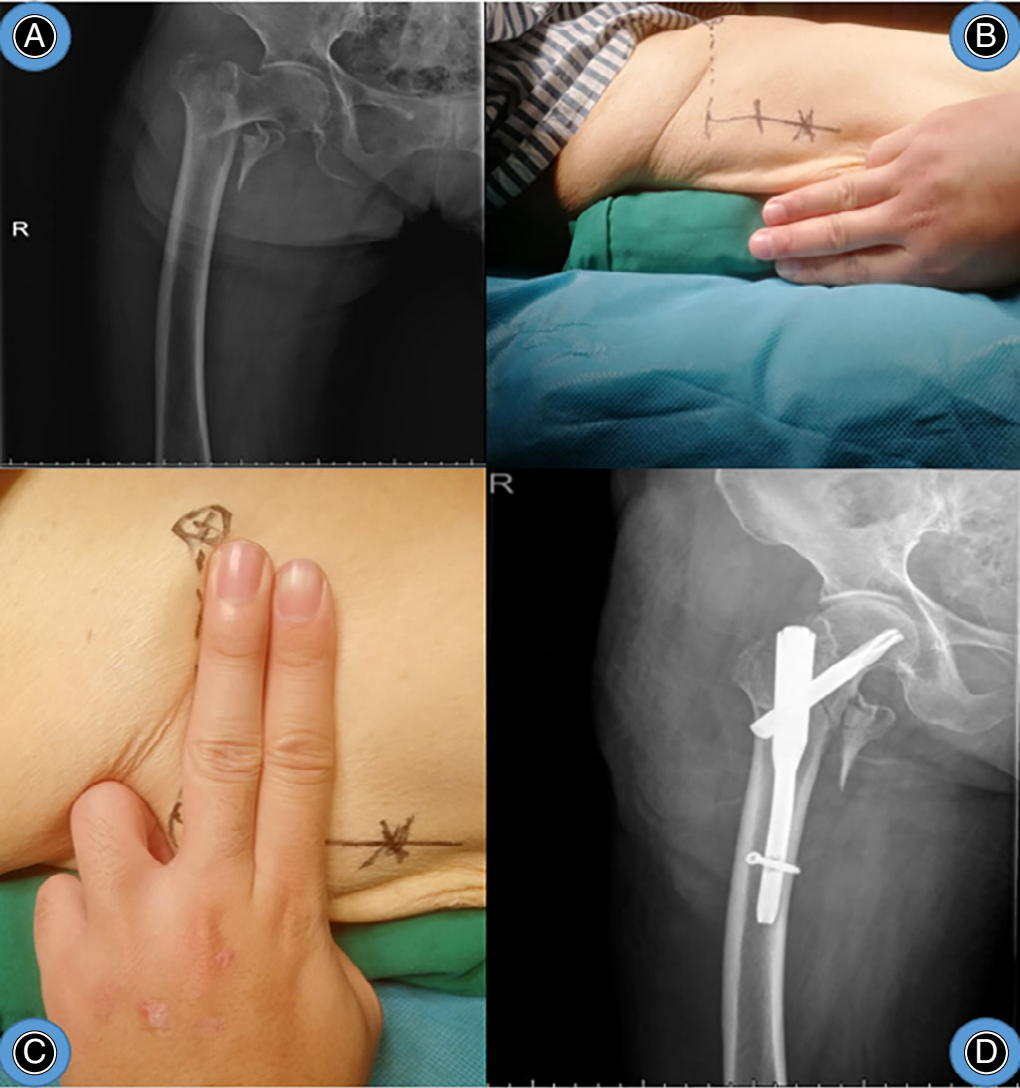
Male, 78 years old, fall injury, right intertrochanteric fracture, treated with “three‐finger method” assisted by PFNA. (A) preoperative X‐ray of pelvis. (B) patient position of 3 fingers high. (C) incision position. (D) postoperative X‐ray of right hip. The patient position and incision position was as same as Fig. 9.

Typical cases are shown in Figs 8, 9, 10.

## Discussion

Fractures from the base of the femoral neck to the upper part of the lesser tuberosity are called intertrochanteric fractures (ITFs). Like most hip fractures, it is most common to see greater trochanter impingement after a lateral fall^29^. Conservative treatment for ITFs require lying in bed for a long period of time; with this treatment, skin traction of the limb can easily lead to a series of complications, such as hip pronation, limb shortening, muscle atrophy, nerve compression, and paralysis. In the elderly population, which is associated with high blood pressure, heart disease, diabetes and other chronic diseases, lying in bed for a long time can often aggravate the condition and lead to morbidities, such as: lung and blood clots in the brain, heart, kidney, and lung; urinary tract infections; and pressure sores^12^. Therefore, the present and consistent view is, for elderly patients who can tolerate such procedures, to operate as soon as possible and to perform early activities in bed in order to reduce complications, reduce the risk of death, and improve the quality of life of patients with fractures^30^. The British NICE hip fracture clinical guidelines suggest that elderly patients should undergo surgery within 48 h as often as possible in order to reduce mortality^31^.

### *The “Three‐Finger Method” Assisted by PFNA Surgery and its Advantages*

PFNA surgery is popular at home and abroad and is favored by orthopaedic surgeons. It has become the mainstream for the clinical treatment of ITFs. To simplify the operation, shorten the operation time, improve the operation efficiency, and better promote the recovery of patients, we improved the PFNA surgery and adopted the “three‐finger method” for assistance. In other words, we used the index finger, middle finger, and ring finger as assistance during the operation.

**Figure 11:**
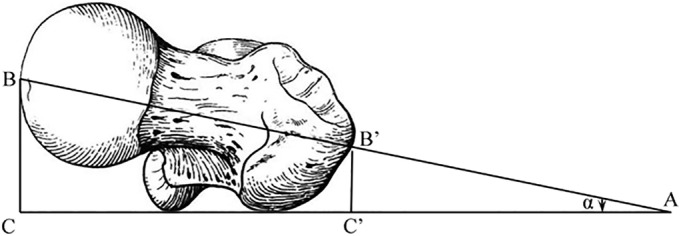
The relevant data according to the calculation of the angle of declination.

Firstly, to facilitate the operation, we ensured that the affected side of the hip joint was elevated and offset the femoral neck angle for declination. We needed to pad the affected hip, but there is no standard for pad height. According to the measurement calculation (Fig. 11), we applied three fingers to simplify the operation steps, shorten the operation time, and improve the operation efficiency. In general, the average sum of the transverse diameters of the index finger and middle finger is 3 cm, and the average sum of the transverse diameters of the index finger, middle finger, and ring finger is 5 cm. Due to the fact that the average of the femoral head and neck shaft length “BB” is 9.34 cm, and that the average upper and lower diameter of the femoral neck stem junction “b” is 3.63 cm, the femoral neck has an average 15°–20° of declination^32^, Sin17°≈0.292, B'C'≈1/2b≈1.82 cm, BC/AB=B'C'/AB'=Sinα≈sin17°, namely, the BC≈AB ×0.292, AB'=B'C'/ Sinα≈1.82/0.292≈6.23 cm, AB=BB'+AB'≈15.57 cm, namely, BC×0.292≈5 cm. Considering the muscle and adipose tissue, the hip pad height is approximately 5 cm, that is, the height of three fingers, which offsets the femoral neck angle of declination to 15°–20°. We can thus insert the guide needle into the femoral neck roughly parallel to the operating table to avoid the problem due to the angle of declination by repeatedly adjusting the needle, loosening the femoral head cervical bone, and not firmly fixing the screw blade.

When selecting the surgical incision, we placed the two fingers of the index finger and middle finger together. The index finger touched the anterior superior iliac spine and was moved backward, vertically, along the longitudinal axis of the femoral shaft. A longitudinal incision, approximately 3 cm in length, was made at the width of two fingers. In obese patients, or in patients with comminuted fractures of the greater trochanter, the greater trochanter is not easily accessible. When we used the greater trochanter to mark the incision, there was often a deviation, thus wasting time, resulting in the incision being too long, increasing the trauma, increasing the difficulty of surgery, and affecting the operation. We used the index finger and middle finger to mark the anterior superior iliac spine with a clear position, a small incision, a small amount of trauma, and a reasonable location. With the expansion of the medullary cavity and insertion of the main nail, which was not blocked by the skin, the result was a convenient operation and an improvement in the surgical efficiency. This procedure is ideal for beginners, as it is simple and practical and has wide clinical applications.

When determining the position of the insertion point of the greater trochanter, we used the index finger to accurately select the insertion point, assist the insertion of the guide needle, and position the needle accurately, thus avoiding damage caused by repeated operations, shortening the operation time, and improving the operation efficiency.

The whole process of the “three‐finger method” is simple, that is, according to the sequence of “3‐2‐1,” which is easy to remember and perform. It greatly simplifies the operation process, shortens the operation time, improves the accuracy of the operation, and improves the operation efficiency, which can be widely promoted in clinical practice.

### *Outcomes of the “Three‐Finger Method” Assisted by PFNA Surgery*

PFNA is designed with a spiral blade. By tapping into the operating mode and using the large surface area of the blade to obtain the pressure effect of the bone, this procedure effectively avoids the destruction of the femoral head, increases the structural strength of the femoral head and neck, avoids the collapse of the femoral head, and lowers the incidence of femoral head necrosis^26^, ^33^. We followed up with 123 patients, and only one had femoral head necrosis; the necrosis rate was only 0.8%. On the one hand, a low femoral head necrosis rate is associated with the design of the helical blade; on the other hand, it is associated with the broken part of the blade. Of course, elderly patients cannot be excluded because of their low activity level, which results in a low amount of weight‐bearing on the femoral head and a low pressure on the femoral head; this results in a low incidence of femoral head necrosis, which requires follow‐up analysis of large samples in the future. In addition, after the spiral blade is inserted into the femoral head and neck, the blade cannot rotate and the bone anchor closes tightly with the greatest degree of bone filling pressure and anchorage force, which is not easy to loosen, with the greatest degree of bone filling pressure and anchor force^34^; thus, this procedure is more suitable for patients with fractures due to osteoporosis or instability and is advantageous for patients who participate in early weight‐bearing^35^. This procedure can improve that curative effect. After our long‐term follow‐up, we found no loosening of the spiral blade or perforation of the femoral head.

The PFNA operation can alleviate pain. On the one hand, this outcome is due to the small surgical trauma, the fixation of the fracture, the quick recovery of the fracture, the rapid recovery of the patient, and the low incidence of pain. On the other hand, elderly patients cannot be excluded from the pain response due to their low activity levels, low levels of irritation to the femoral head, and the low incidence of pain. Based on the long‐term follow‐up results, we found that the functional recovery of the hip was associated with patient age; the older the patient was, the worse the recovery of hip function. There was no direct relationship with PFNA surgery itself, mainly due to the patient's age. The body's own activity decreased, resulting in a decline in the function of the hip joint. Among the 123 patients who were followed, three showed wound exudation. We considered that these patient were older, with relatively poor nutritional status and low levels of protein consumption, which had nothing to do with the operation itself.

In summary, patients with intertrochanteric fractures were followed for up to 8 years to observe hip joint pain, and functional recovery outcomes and complications were also recorded. We found that the “three‐finger method” used to assist PFNA for the treatment of intertrochanteric fractures of the femur simplified the surgical procedure, greatly reduced the surgical difficulty, shortened the operation time, and improved the surgical efficiency. Its complication rate was low, its long‐term curative effect was good, it is easy to learn and master, and it is worth implementing in the clinic.

### *Study Limitations*

This study is only a summary of our experience of “three‐finger method” assisted by PFNA to treat intertrochanteric fracture without comparison with traditional method. For the next step, a control group should be set up, detailed pictures of the surgery should be taken, and the variate analysis of functional outcomes should be further investigated so as to better illustrate the advantages of our method.
